# Nurses’ awareness of the availability of HIV and AIDS research

**DOI:** 10.4102/hsag.v28i0.2200

**Published:** 2023-03-23

**Authors:** Justin O. Rojaye, Robert T. Netangaheni

**Affiliations:** 1 Department of Health Sciences, Faculty of Human Sciences, University of South Africa, Tshwane, South Africa

**Keywords:** Awareness, healthcare research, HIV and AIDS, nurses, participation

## Abstract

**Background:**

Hardship of human immunodeficiency virus (HIV) and acquired immunodeficiency syndrome (AIDS) transmission in Nigeria needs research-based strategies that scientifically inform research findings for relevant research advocacy.

**Aim:**

This study aimed to enhance nurses’ awareness of the availability of HIV and AIDS research in Nigeria.

**Setting:**

The research was carried out in a general hospital, a tertiary hospital in Ijebu Ode, Ogun State, Nigeria.

**Methods:**

A qualitative technique was used, as well as an exploratory-descriptive design was employed. Semi-structured interviews were carried out. Three focus group interviews with seven participants in each group were used to gather data.

**Results:**

This study showed the value of nurses’ awareness of the availability of HIV and AIDS research in overcoming obstacles to successfully controlling HIV and AIDS transmission in a hospital context in Nigeria. The study results demonstrated the nurses’ understanding of local and global research. The result of the study also demonstrated the essential role of collaboration with partners in enhancing nurses’ awareness of the availability of HIV and AIDS research in Nigeria.

**Conclusion:**

The study found that nurses in Nigeria were usually unaware of their role in healthcare research development in the context of HIV and AIDS. They preferred to go with the flow of events.

**Contribution:**

This study affirms the importance of nurses’ awareness of the availability of HIV and AIDS research in overcoming obstacles to successfully controlling HIV and AIDS transmission in a hospital context in Nigeria.

## Introduction

Despite the fact that Africa constitutes just 11% of the world’s population, Africa remains the epicentre of the human immunodeficiency virus (HIV) and acquired immunodeficiency syndrome (AIDS) pandemic (Avert 2017). Unfortunately, Nigerian nurses are still not fully aware of the importance of research on the transmission of HIV and AIDS. Therefore, it is very important to develop strategies to enhance the nurses’ awareness of HIV and AIDS research in the prevention and treatment of HIV and AIDs in Nigeria. According to the World Health Organization (WHO 2017) guidelines on HIV and AIDS screening, treatment for those living with HIV should provide them with a complete package of services inclusive of screening and diagnosis, treatment, prophylaxis and preventive treatment for specific pathologies, such as tuberculosis, as well as rapid initiation of antiretroviral therapy [ART] and adherence support to minimise morbidity and mortality. In Nigeria, about 3.4 million people are infected with HIV and AIDS (Avert 2017). At the same time, approximately 217 000 people are killed by AIDS annually (Omowaleola 2018). However, progressive HIV programmes have resulted in a 43% decrease in HIV infections worldwide between 2003 and 2011 (Avert 2017).

The healthcare industry plays a crucial role in the response to HIV, with nurses seen as prominent policymakers in the health sector through their engagement in healthcare research development (Ogbolu et al. 2018). Therefore, a research participation orientation for nurses has the potential to be a systematic way of addressing obstacles to the effective engagement of nurses in evidence-based healthcare research development in HIV and AIDS transmission programmes (Rivaz et al. 2017).

Based on Sturke et al. (2018) assertions, it is evident that nurses, who are vital implementers of HIV policies, ought to be actively engaged in healthcare research creation to obviate HIV research implementation as the sole preserve of nurses and management. Moreover, Richter et al. (2017) also support the argument that nurses should be included in creative research approaches in the initiatives against HIV pandemic. In this regard, the South African Department of Health (DoH) (2013) has credited the high success rate in HIV programme coverage to nurses’ vital role in executing preventative techniques.

### Problem statement

In addition, nothing is known regarding nurses’ awareness of HIV and AIDS research availability in the prevention and treatment of HIV and AIDS in Nigeria. There is a gap between research creation by nurses and attempts for successful implementation in the context of nurses’ awareness of the availability of HIV and AIDS research in Nigeria. This project will illustrate techniques for increasing nurses’ awareness of the availability of HIV and AIDS research to improve patient care quality. Nurses providing care to HIV-positive patients must respect their dignity, confidentiality, rights and choices which could be achieved through the awareness of research that speaks about all these procedures. As in other locations, nurses offer first-line care to patients living with HIV and AIDS, but they are not heavily engaged in generating HIV-related health research (Richter et al. 2017). This is seen as a significant gap between research and practice in health education and research. It is empirical to incorporate nurses in developing healthcare research to suit the needs of their HIV and AIDS patients. Improving knowledge, communication and information sharing, and the availability of necessary resources are critical in safeguarding healthcare personnel and HIV-infected patients (Richter et al. 2017). Their judgemental behaviour, stigma and subsequent discrimination, and noncooperative create gap in awareness of research availability.

### Study purpose

This study aimed to explore and describe the nurses’ awareness of HIV and AIDS research availability in Nigeria. The study also sought to develop strategies to enhance the nurses’ awareness of HIV and AIDS research to meet the needs of HIV and AIDS patients. The study intended to find answers to the following research questions:

- How do nurses aware of HIV and AIDS research availability in Nigeria?
- What research strategies could be employed to engage nurses in the awareness of the availability of HIV and AIDS research?

## Research design and method

This research used a qualitative, exploratory and descriptive approach. The qualitative setup was a method for comprehending and gaining insight into a situation, society, person and event under investigation (Silverman 2019). The descriptive design focuses on how and why questions to paint a picture of a particular circumstance, social scene and connections (Silverman 2019). This dynamic method employs intuitive and inductive ways to obtain a more in-depth grasp of what has been studied and constantly enhance interpretations (Creswell 2018). Researchers rely on their past knowledge of research settings, participants and records (Taylor & Bogdan 2018). According to Hatch (2022), qualitative analysis is the process of organising and probing data for researchers to recognise patterns, uncover themes, establish connections, generate explanations, provide interpretations, issue critiques and construct ideas. The everyday tasks include synthesis, assessment, interpretation, categorising, hypothesising, comparing and pattern identification (Hatch 2022).

### Setting

The research was carried out at a hospital in Ijebu Ode, Ogun State, Nigeria. The Ijebu Ode General Hospital is a referral hospital. The hospital is one of the oldest hospitals that manage all medical and surgical patients. The hospital is the largest in the Ijebu area and has about 250 beds.

### Population

The target population consisted of registered nurses working full-time at the hospital.

### Sampling and sample method

The purposive sampling approach was employed to recruit participants who participated in the implementation and development of the research. The researcher included nurses who are working in HIV units and with experience of people living with HIV and AIDS. Student nurses and other medical personnel were excluded from this study. The participants were asked to volunteer and share their experiences and lessons learned. The study’s sample included 21 nurses. The goal was to choose individuals who were informed about HIV and AIDS and could offer rich data on the availability of HIV and AIDS research. There were three focus groups, each with seven individuals.

### Data collection and tools

The researcher used a semi-structured focus group interview guide with open-ended questions to obtain detailed information regarding nurses’ awareness of HIV and AIDS research in Nigeria. The data-gathering process was divided into different steps, each of which helped to form the study’s conceptual framework. Firstly, data were obtained in 2022 utilising a semi-structured interview, implying that it would let participants discuss their thoughts on the availability of HIV and AIDS research in Nigeria. Focus groups are appropriate when participant engagement has the potential to give broader insights that may be developed via discussion (Creswell 2018). The benefit of focus groups is that members’ perspectives may be contrasted. During the interviews, a scribe took notes and no digital recordings were made. The main questions that directed the interviews were: How are nurses aware of HIV and AIDS research availability in Nigeria? and What research strategies must be developed to engage nurses in the awareness of HIV and AIDS research? This was followed by probing inquiries into the study’s aims. Data collection continued until no new information was discovered, indicating that the data were saturated. The focus group interviews lasted for about an hour in a quiet place during working hours. The researcher was the main conductor of the interview. The researcher was granted permission to record the interviews.

### Data analysis

According to Creswell (2018), data analysis starts with data management, reading and memoing, summarising, categorising, analysing and producing a narrative that correctly depicts the case study’s story. Furthermore, Creswell (2018) asserts that participants’ interpretations are critical in offering the best explanations for their actions, behaviours and ideas in qualitative analysis. Consequently, theme analysis was selected as the approach for this research because it is both simple and theoretically flexible (Alhojailan 2022). Furthermore, this step ensures that the themes are correctly associated with the data (Patton 2019).

Data analysis was carried out by recordings of interviews taken during data collection and transcribed verbatim. Data analysis was placed in tandem with data gathering. The researcher produced information by gathering data in specific segments. For the proposed study, data were collected via audio recordings that were transcribed and organised under the main themes identified by the research questions the study seeks to answer. The researcher used Creswell’s (2018) approach for analysing focus group interview conversations, which consists of four steps: transcription from audio, data coding, data interpretation and data reporting. Firstly, each interview transcript was read and re-read to obtain a broad idea. Secondly, the transcript was carefully examined to find key information. In addition, the researcher cross-checked the transcripts against the original recordings of the interview audio data to confirm correctness (Silverman 2019).

The researcher sought out linkages within the data that created a broader picture. Data analysis helps identify emerging arrangements or themes that clarify the subject of study (Williamson, Given & Scifleet 2018). In this research study, the information derived from focus group discussions was transcribed and organised into the theme for presentation using the modified six phases of data analysis outlined by Creswell (2018) as cited in Allen Jensen and Laurie (2017).

### Trustworthiness

The level of trust in the facts, interpretation and procedures employed to assure research quality is called trustworthiness (Polit & Beck 2021). Credibility, transferability, dependability and confirmability were examined to establish the study’s trustworthiness. Member verification was used to establish credibility (Brown & Schmidt 2017). During member verification, the researcher shared the data with the participants to ensure correctness and coherence with their experiences. The researcher also gained credibility by offering a complete account of the procedures and techniques used in the study. Transferability was achieved by adequately defining the study’s context and evaluating the data’s representativeness. Representativeness was obtained by choosing a diverse group of participants who provided rich, all-encompassing data to identify saturation levels. As a result, eligible registered nurses were selected to participate in the interviews. Dependability was accomplished by establishing a clear and thorough explanation of the procedures during data collection so that other reviewers could validate the outcomes. The impartiality of research throughout data collection and analysis is referred to as confirmability (Creswell 2018). Confirmability was achieved by accurately and thoroughly documenting the processes, procedures, themes and sub-themes.

### Ethical considerations

The College of Human Sciences Research Ethics Review Committee (CREC) of the University of South Africa approved the research (reference number: 60825588_CREC_CHS_2022). This study followed the Code of Ethics for conducting research with human subjects. The hospital authorities and all the participants consented in writing. The participants were also guaranteed anonymity and confidentiality. Therefore, there was no probability of risk and/or benefit occurring as a result of participation in this research study.

## Results

Data were collected from a sample of 21 nurses who have knowledge and experience in the treatment and management of patients with HIV and AIDS. The interview data yielded one theme and six sub-themes (Table 1).

**TABLE 1:** Nurses’ awareness of the availability of human immunodeficiency virus and acquired immunodeficiency syndrome research.

Themes	Subthemes
Nurses’ intimate understanding of healthcare research	Nurses’ understanding of global research Nurses’ understanding of local research Barriers to HIV and AIDS research development Implementing local and global research The nurses’ roles in HIV and AIDS research The essential role of collaboration with partners

HIV, human immunodeficiency virus; AIDS, acquired immunodeficiency syndrome.

### Theme 1: Nurses’ intimate understanding of healthcare research

The participants’ intimate understanding of healthcare research refers to their in-depth understanding of the consequences of HIV and AIDS on the healthcare system. The sub-theme sheds light on the degree to which nurses are aware of the trends, impacts, ameliorative techniques and accompanying impediments. Clinician scientists, particularly nurse researchers, play a critical role in providing research findings that may be used to guide and enhance healthcare practice, education and policy. Health research is often carried out using one of three paradigms: quantitative, qualitative and hybrid techniques. The contributions of all three focus groups are denoted in quotes as N1–N21, with N1 referring to participant 1 up to participant 21. As a result, the participants’ HIV and AIDS research awareness was condensed into a single theme. Table 1 shows the theme and subthemes.

#### Subtheme 1: Nurses’ understanding of global research

The focus group members discussed interpreting global HIV and AIDS research. In Nigeria, HIV and AIDS are regarded as major public health issues. One survey participant, for example, pointed out that HIV and AIDS had been a difficulty for the health system and the general population. This participant agrees while stating that the impact of HIV is most visible in society’s most vulnerable people. Furthermore, HIV has become a perpetual and ingrained concern because of its negative consequences for future generations. The following are actual quotes that support these arguments:

‘I know through research that HIV was once a worldwide concern, but it is now mostly a problem in our local government areas nationwide.’ (N1, female, 34 years)

‘According to information from the internet, we were able to know what HIV and AIDS worldwide prevalence. It was stated that around 35 million persons are infected globally. Still, more than 90 percent of those infected live in developing countries, with Sub-Saharan Africa accounting for two-thirds of the global prevalence. So, you can see that it is not a worldwide issue; it may be a global worry, but it is more of a developing-country problem or call it Africa’s problem.’ (N2, female, 28 years)

‘We were able to know that HIV was first found in the state in 1987 via worldwide research, but it was difficult to convince many of the reality of such a disease, and the concept of mother-to-child HIV transmission remains a myth to many.’ (N3, female, 29 years)

According to MacLean (2021:2), the supply and demand principles impact the worldwide research perspective. The capacity to deliver research results is a vital business for knowledge producers such as universities and research institutions. Indeed, research results constitute a kind of assessment. Rapid advancements in information technology have expanded data availability to the point that practically everyone with a mouse and Internet connection may function as a quasi-researcher (MacLean 2021:1). Recognising global HIV and AIDS trends is the first step in ensuring that patients with HIV and AIDS are appropriately managed. It is challenging to examine and treat HIV and AIDS if they are not recognised (Rasmi Issa, Awaje & Khraisat 2017:10).

#### Subtheme 2: Nurses’ understanding of local research

Participants displayed an awareness of the many strategies implemented by their organisation to tackle the threat of HIV and AIDS. Unfortunately, there is very little local research on HIV and AIDS transmission in Nigeria (Ogbolu et al. 2018). The initial local strategy, for example, was to create an HIV coordinating agency known as the National Agency for Control of AIDS (NACA), followed by state and municipal government bodies. The following are actual quotes that back this up:

‘I think this organisation [*the State AIDS Control Agency*] was the first local purposeful attempt since we continued trying different methods without coordination of actions.’ (N6, female, 50 years)

‘A method of integrating HIV and AIDS prevention into the health care system is to guarantee that local services are available for every pregnant woman, regardless of where she is, throughout the state.’ (N4, female 52 years)

‘I believe HIV and AIDS transmission is real and local people need to understand it.’ (N1, female, 34 years)

‘Many people are more aware of it and trying to prevent it, but the belief of local people is still a problem.’ (N7, female, 45 years).

‘The transmission of HIV and AIDS is now more common in rural areas than urban because of self-awareness.’ (N8, female, 52 years)

Another local research plan mentioned by participants was the state HIV and AIDS operational plan prepared by all stakeholders; the plan gives guidance on how to incorporate research development into nursing practice and recognises the role of various healthcare stakeholders. Participants also stated that the state’s successful technique includes integrating HIV and AIDS research development into all places where health services are provided. Human immunodeficiency virus and AIDS services are available at all levels of healthcare in the state.

#### Subtheme 3: Barriers to HIV and AIDS research development

This institution’s participants also showed knowledge of HIV and AIDS research implementation difficulties. Participants discussed the obstacles to research implementation, such as cultural attitudes and a lack of human resources. For example, according to one participant, many individuals still choose traditional birth attendants for health treatment. These hurdles include a lack of service uptake, poor adherence to treatment, a lack of agreement from a spouse or family member to treatment and superstition about HIV. In addition, misconceptions about HIV persist in communities, with some pregnant women refusing treatment after testing positive. The following are actual quotes that back this up:

‘One major barrier to implementing HIV and AIDS research was because many nurses do not believe in the reality of HIV and AIDS as a sickness; others think witches cause it.’ (N10, female, 44 years)

‘Another difficulty was that they were positive after some women were contacted. They stopped attending the hospital and reverted to their churches or traditional birth attendants, making it impossible to track these females down.’ (N5, male, 45 years)

‘Some customers conceal that they need to take antiretroviral medications because they are afraid their spouse will kick them out of the house.’ (N11, female, 52 years)

Participants proposed numerous strategies for nurses’ awareness of the availability of HIV and AIDS research challenges. In addition, participants described how various strategies could be implemented to achieve good outcomes. According to Laprise and Bolster-Foucault (2021:1), efforts to improve access to HIV and AIDS research implementation should consider barriers and facilitators at the individual, healthcare provider and policy levels focusing on research services accessibility, inclusivity, convenience and confidentiality (Laprise & Bolster-Foucault 2021:1).

#### Subtheme 4: Implementing local and global research

According to the participants, creative, community-driven solutions are vital to eliminating the HIV epidemic in Nigeria. According to them, Nigeria funded priority jurisdictions (state and local health departments) to develop innovative, locally tailored plans that lay the groundwork for scaling up the initiative’s critical strategies in a way that addresses unique regional needs, reduces health disparities and achieves health equity in each community. The following are actual quotes that back this up:

‘Every nurse understands the importance of community nursing in eradicating HIV and AIDS in Nigeria but fails to implement local and global research when caring for HIV patients.’ (N9, female, 36 years)

‘The key to HIV and AIDS local and global research implementation is to provide HIV and AIDS plans to the grassroots.’ (N15, female, 47 years)

‘The government should establish additional local healthcare units in each local government to cope with implementing HIV and AIDS research.’ (N13, female, 56 years)

According to Laprise and Bolster-Foucault (2021:1), integrating the awareness of local and global research on HIV and AIDS is a gateway to HIV treatment and prevention. Furthermore, this lack of awareness prevents the HIV and AIDS research development, as well as some key aspects that may be harnessed to accelerate HIV and AIDS development (Laprise & Bolster-Foucault 2021:1). It entails the ongoing participation of nurses and broad-based communities in identifying methods to strengthen the awareness of HIV and AIDS research within their jurisdiction to achieve the HIV and AIDS initiative (Laprise & Bolster-Foucault 2021:1).

#### Subtheme 5: The nurses’ roles in HIV and AIDS research

Several participants say nurses have a unique role in Nigeria’s HIV and AIDS research development. Therefore, eradicating HIV and AIDS depends more on nurses’ expertise in research development. In addition, participants reported that each active health institution must have at least one nurse knowledgeable about HIV and AIDS research development to organise operations and deliver standard HIV and AIDS services. The following are actual quotes that back this up:

‘Nurses are essential to the state’s HIV and AIDS responses.’ (N12, female, 45 years)

‘We have nurses in charge of some of these places.’ (N16, female, 47 years)

‘As long as there is a qualified nurse at the institution, we are certain that excellent care will be delivered, including antiretroviral antibiotic control.’ (N14, female, 38 years)

Nurses have a unique role in assisting the Nigerian government in effectively implementing nationally approved HIV and AIDS programmes to eliminate HIV in the state. As a result, nurses’ critical involvement in completing research towards the eradication of HIV and AIDS is very important for the prevention of transmission of HIV and AIDS in Nigeria. Furthermore, according to Koenig (2019:1), rapid changes happening domestically and globally, including transformations in demography, languages, epidemiological patterns and social systems that directly affect patient treatment.

#### Subtheme 6: The essential role of collaboration with partners

Most participants agreed that cooperation with other healthcare partners in creating awareness of the availability of HIV and AIDS research is critical to eradicating HIV and AIDS in Nigeria. Participants mentioned that they work with physicians, counsellors, spiritual leaders and psychologists to satisfy the health requirements of their HIV and AIDS patients. The participants claimed that eliminating HIV and AIDS in Nigeria would be difficult without the involvement of partners to create and develop HIV and AIDS research. The following are actual quotes that back this up:

‘At the start of care, several partnerships meetings with physicians and patients to establish the kind of patient treatment always place.’ (N17, female, 45 years)

‘Nurses usually suggest that all HIV and AIDS patients see a psychologist.’ (N20, female, 53 years)

‘Nurses assist patients in receiving emotional therapy by talking with their pastors or other religious leaders of choice.’ (N21, female, 44 years)

‘Because counselling is a crucial component of therapy for any HIV and AIDS patient, nurses usually recommend patients to visit a counsellor.’ (N19, female, 44 years)

‘The federal government collaborates with each target jurisdiction to build local HIV prevention programs.’ (N18, female, 37 years)

According to Monaco et al. (2021:1), multi-stakeholder partnerships can promote nurses’ awareness of the availability of HIV and AIDS research in the prevention and patient care changes. To create effective nurses’ awareness of the availability of HIV and AIDS research, there must be intra- and inter-communication among all nurses in developing clinical research. In addition, a comprehensive nurses’ awareness of HIV and AIDS research would create a response to HIV and AIDS prevention that would improve patient outcomes by providing strategic, scientific and economic support (Monaco 2021:1).

## Discussion

The study explored and described the nurses’ awareness of HIV and AIDS research availability in Nigeria. The setting and context in which nurses work provide a once-in-a-lifetime chance to understand the effect of HIV and AIDS on healthcare institutions and the general public. This is because registered nurses in this context deliver person-centred care in their communities. In addition, they oversee the majority of primary healthcare institutions. Therefore, they are aware of developing diseases’ effects on persons in the community and the many health institutions they serve. This finding is compatible with Ndikom and Onibokun (2019), who agreed that nurses’ expertise in creating awareness of HIV and AIDS research availability is created due to their experiences delivering healthcare in each context.

Nurses must be completely aware of the effects of HIV on a compromised healthcare system and their opinions are similar to those of El-Jardali & Lavis (2018). Nurses asserted that chronic illnesses such as HIV and AIDS greatly influenced the health system, depleting its limited human and material resources. According to Osain (2019), the Nigerian healthcare system is insufficient in dealing with chronic and emergent illnesses, which is aggravated by a lack of nurses’ awareness of the availability of HIV and AIDS research and dismal quality of treatment. Furthermore, Avert (2017) observed that if nurses continue to lack awareness of the availability of HIV and AIDS research, Nigeria’s HIV and AIDS programme is at risk, with various obstacles obstructing the fulfilment of established targets even after it was decentralised from tertiary to secondary and primary healthcare institutions.

Nigerian nurses are often well-versed in HIV and AIDS treatment programmes but are lacking in HIV and AIDS research development. Consequently, without a grasp of HIV and AIDS research awareness by nurses, Nigeria’s 32% HIV coverage would not have been conceivable. This study is similar to International Center for AIDS Care and Treatment Programs (ICAP) (2018) findings, which indicated that eradicating HIV and AIDS would be difficult without nurses because they understand the people they serve and can adapt suggested techniques to their local environment. The authors also mentioned that registered nurses teach and supervise lay workers in implementing HIV and AIDS programmes demonstrating that they are well-versed in these techniques but lack awareness of HIV and AIDS research availability.

The role of nurses in awareness of the availability of HIV and AIDS research is consistent with Omowaleola (2018) views that Nigerian registered nurses must play a critical role in accomplishing HIV and AIDS preventive objectives through their awareness of the availability of HIV and AIDS research. Furthermore, Ndikom and Onibokun (2019) revealed that Nigerian registered nurses understood HIV and AIDS techniques such as antiretroviral medications, voluntary counselling and testing, caesarean delivery and new-born feeding alternatives but Nigerian nurses are lacking in awareness of research to back these practices.

## Conclusion

This qualitative study’s findings have described the nurses’ awareness of the availability of HIV and AIDS research in Nigeria. Overall, all three focus group participants felt that participating in the study allowed them to develop the capacity and capability to implement HIV and AIDS research. They also believed that uncovering the elements at work in research was essential to promote the successful delivery of HIV and AIDS research.

## Limitation

This study examined nurses’ awareness of HIV and AIDS research availability in Nigeria. The study’s shortcoming was that it did not address other research elements of nursing practice. However, trustworthiness and ethical consideration were applied to reduce the potential influence of the constraint on the study’s richness. In addition, because of the sample size, the study’s findings cannot be extrapolated to larger groups or populations. For example, if the researcher had used a broad sample covering a vast geographical region, the researcher would have received various perspectives on the study issue.
